# On the Use of Carbon Cables from Plastic Solvent Combinations of Polystyrene and Toluene in Carbon Nanotube Synthesis

**DOI:** 10.3390/nano12010009

**Published:** 2021-12-21

**Authors:** Alvin Orbaek White, Ali Hedayati, Tim Yick, Varun Shenoy Gangoli, Yubiao Niu, Sean Lethbridge, Ioannis Tsampanakis, Gemma Swan, Léo Pointeaux, Abigail Crane, Rhys Charles, Jainaba Sallah-Conteh, Andrew O. Anderson, Matthew Lloyd Davies, Stuart. J. Corr, Richard E. Palmer

**Affiliations:** 1 Energy Safety Research Institute, Swansea University, Bay Campus, Swansea SA1 8EN, UK; alihe@chalmers.se (A.H.); 748963@Swansea.ac.uk (T.Y.); V.S.Gangoli@Swansea.ac.uk (V.S.G.); 958003@Swansea.ac.uk (I.T.); 9901886@Swansea.ac.uk (G.S.); 2124215@Swansea.ac.uk (L.P.); 910979@Swansea.ac.uk (A.C.); 825585@Swansea.ac.uk (J.S.-C.); 934622@Swansea.ac.uk (A.O.A.); 2 Chemical Engineering, Faculty of Science and Engineering, Swansea University, Bay Campus, Swansea SA1 8EN, UK; 3 TECNALIA, Basque Research and Technology Alliance (BRTA), Alava Science and Technology Park, Leonardo da Vinci 11, 01510 Vitoria-Gasteiz, Spain; 4 Nanomaterials Lab, Mechanical Engineering, Faculty of Science and Engineering, Swansea University, Bay Campus, Swansea SA1 8EN, UK; Yubiao.Niu@Swansea.ac.uk (Y.N.); 906934@Swansea.ac.uk (S.L.); R.E.Palmer@Swansea.ac.uk (R.E.P.); 5 SPECIFIC, Materials Science and Engineering, Faculty of Science and Engineering, Swansea University, Bay Campus, Swansea SA1 8EN, UK; R.Charles@Swansea.ac.uk (R.C.); M.L.Davies@Swansea.ac.uk (M.L.D.); 6 School of Chemistry and Physics, University of KwaZulu-Natal, Durban 4041, South Africa; 7 Department of Cardiovascular Surgery, Houston Methodist Hospital, Houston, TX 77030, USA; sjcorr@houstonmethodist.org; 8 Department of Bioengineering, Rice University, Houston, TX 77005, USA; 9 Department of Biomedical Engineering, University of Houston, Houston, TX 77204, USA; 10 Swansea University Medical School, Institute of Life Science 2, Swansea University, Singleton Park, Swansea SA2 8PP, UK

**Keywords:** carbon nanotube, plastic, chemical recycling, life cycle assessment, ethernet, circular economy, data transmission, carbon footprint

## Abstract

For every three people on the planet, there are approximately two Tonnes (Te) of plastic waste. We show that carbon recovery from polystyrene (PS) plastic is enhanced by the coaddition of solvents to grow carbon nanotubes (CNTs) by liquid injection chemical vapour deposition. Polystyrene was loaded up to 4 wt% in toluene and heated to 780 °C in the presence of a ferrocene catalyst and a hydrogen/argon carrier gas at a 1:19 ratio. High resolution transmission electron microscopy (HRTEM), scanning electron microscopy (SEM), thermogravimetric analysis (TGA) and Raman spectroscopy were used to identify multiwalled carbon nanotubes (MWCNTs). The PS addition in the range from 0 to 4 wt% showed improved quality and CNT homogeneity; Raman “Graphitic/Defective” (G/D) values increased from 1.9 to 2.3; mean CNT diameters increased from 43.0 to 49.2 nm; and maximum CNT yield increased from 11.37% to 14.31%. Since both the CNT diameters and the percentage yield increased following the addition of polystyrene, we conclude that carbon from PS contributes to the carbon within the MWCNTs. The electrical contact resistance of acid-washed Bucky papers produced from each loading ranged from 2.2 to 4.4 Ohm, with no direct correlation to PS loading. Due to this narrow range, materials with different loadings were mixed to create the six wires of an Ethernet cable and tested using iPerf3; the cable achieved up- and down- link speeds of ~99.5 Mbps, i.e., comparable to Cu wire with the same dimensions (~99.5 Mbps). The lifecycle assessment (LCA) of CNT wire production was compared to copper wire production for a use case in a Boeing 747-400 over the lifespan of the aircraft. Due to their lightweight nature, the CNT wires decreased the CO_2_ footprint by 21 kTonnes (kTe) over the aircraft’s lifespan.

## 1. Introduction

If carbon nanotubes (CNTs) are to be used for their lightweight and electrical conduction properties on a large/global scale [1], is there a scenario that justifies their use, especially given the (typically) large embodied energy requirements for their manufacture? Moreover, in the age of sudden climatic shifts linked to carbon emissions, then where would the carbon come from that would be used to make these CNTs? Additionally, can the related production method from that carbon source contribute to the goal of achieving a positive climate output? To address these questions, we suggest that both plastics and solvents be used as carbon sources for CNT manufacture, and that they can create a positive environmental impact over the lifespan of their application in the aerospace sector. For instance, one of the key attributes of CNTs is being lightweight with a density that is 1/6th that of copper. This mass decrease will result in fuel savings for the automotive and aviation sectors.

Plastic products synthesised from recycled plastics are often of inferior quality compared to freshly manufactured plastics, and are not feasible for the same applications. This results in fresh plastics having a wider range of uses, and therefore, holding greater economic value. Moreover, the recycling process is often thwarted by the inclusion of fillers, pigments, and flame retardants, for example. Additionally, mixed plastics and/or composite plastic products are also challenging or impossible to recycle unless separated. To overcome these problems, one can consider open-loop recycling by making new products other than plastic. The most prominent technique uses thermal pyrolysis to break down the long chain polymer molecules into smaller, less complex molecules [2] via the application of intense heat [3]. This is typically carried out in the absence of oxygen to avoid the formation of undesirable carbon oxides, and is done in the presence of a catalyst to increase efficiency, tailor the resulting product, and improve scalability. The product mix is typically composed of oils, chars, and gases that require subsequent refinement and separation, thereby requiring more energy. As such, the primary focus of the field has been to improve synthesis techniques with decreased energy requirements and to create more refined and homogenous products [4,5]. To that end, we have developed a novel approach towards the pyrolytic growth of carbon nanotubes by including a dissolution step prior to the high temperature cycle.

CNT growth from plastics is typically achieved using pyrolysis, whereby polymers such as polypropylene (PP) [6], polyethylene (PE) [7], polyethylene terephthalate (PET) [8] and high-density polyethylene (HDPE) [9] are heated in a solid–gas fluidized bed reactor [10]. The resultant off-gas traverses via a carrier-gas to a catalyst site to complete the conversion from vapor to the solid CNT product. The pyrolytic growth of CNTs can be improved by the addition of a thermodynamically compatible solvent [11,12,13] such as toluene [14,15] to dissolve plastics including polystyrene (PS). The dissolution process confers five benefits over dry plastic pyrolysis. Polymer disentanglement initiates in the solvent [16], thus increasing the reactive surface area. The polymer begins to decompose [17], and thus lowers the required energy for subsequent C–H cleavage, thereby facilitating the processing of mixed plastic products. All nonsoluble material crashes out of solution, such as flame retardants and other noncompatible additives, effectively cleaning the plastic prior to CNT growth. Also, once dissolved, mass transport at scale is readily achieved using pumps and pipes; therefore, the liquid injection method is beneficial for large-scale operations. Moreover, mixing plastics in a solvent increases the carbon density, which can lead to increased production capacity of CNTs via chemical recycling of mixed plastics and solvents.

In conducting a life-cycle analysis (LCA), one must consider the lifespan of the material from inception to application, with particular focus on its manufacturing, transformation, use, and disposal [18]. The complexity of the phenomena involved and the interactions among these steps is a source of uncertainty regarding the real value of the impacts, which is why we can only create “potential” life cycle assessments. Although considered for a long time as an experimental tool, the international standards ISO 14040 and 14044 (revised in 2006) have set the methodological and ethical bases for this type of assessment. In this age of climate uncertainty, it is imperative to adopt protocols and solutions that minimise harm compared with the problems of ‘business as usual’, which these protocols are intended to solve [19].

The energy cost associated with the high entropy and exergy of plastics reconstitution is often cited as the single biggest reason to avoid plastics feedstock, especially with respect to CNT growth. Moreover, this reason is used to justify the continued use of virgin hydrocarbons, despite the fact that CNT growth from these sources never accounts for the energy or material cost associate with purification and delivery of the refined feedstocks. Though this is true in terms of energy consumption, the longer-term challenge deals with the supply of virgin materials based on oil extraction before reaching a peak oil scenario. Before that point, it would be prudent to establish and develop the science and technology to use premade plastics, in all the states in which they are found [20,21], especially given that these materials will otherwise be strewn about the planet, ending up in our soil and food supply [22].

Herein, we report that carbon from plastics can act as a feedstock for carbon nanotube growth by the upcycling of plastic to high-value materials via a chemical process. This can be considered a viable alternative to landfill and incineration. Environmental challenges exist from both liquid and solid hydrocarbons, so we applied toluene and PS as model materials.

## 2. Materials and Methods

### 2.1. The Synthesis of Carbon Nanotubes

The growth of multiwalled carbon nanotubes (MWCNTs) was carried out via catalytic chemical vapour deposition (CCVD) in a two-zoned horizontal furnace (Nanotech Innovations SSP-354, Oberlin, OH, USA) liquid injection reactor (LIR), with full details described previously [23]. In summary, control CNTs were grown by injection of 1 mL (865 mg) anhydrous toluene (98% (C_6_H_5_CH_3_) Sigma Aldrich (Gillingham, UK)) at 5 mL/h under a gas flow of 1 L/min using blended carrier gas having 5 vol% hydrogen in argon (BOC, Guildford, UK) into the two-zone horizontal furnace. The first zone, used for vapour formation was set to 225 °C; the second zone used for growth was set to 780 °C. MWCNTs were grown in a 100 cm long quartz tube with diameter of 38 mm (Multi-Lab, Newcastle upon Tyne, UK). All reactions were carried out using a 20 gauge needle.

Polystyrene (C_8_H_8_)_n_, with a molecular weight of 6400 (Mn 64,000, Sample#P2444-S, Polymer Source Inc., Dorval, QC, Canada), was added to toluene in concentrations of 1, 2, and 4 wt% (*w*/*w*) using PS masses of 8.75, 17.5, and 35.00 mg, respectively. All reactions were carried out with a fixed catalyst ratio of ferrocene (5 wt% *w*/*w*) (98% (C_10_H_10_Fe) Sigma Aldrich (Gillingham, UK)) with respect to the total reactant from toluene and/or toluene and polystyrene. Prior to each growth, the reactants were thoroughly mixed and degassed for 15 min using bath sonication. All materials were used as received without prior cracking or drying, and handled as described here [24]. Each concentration of PS and control was grown three times to ensure that the observed trends were valid for each series. No noticeable effect or carbothermal reduction from the aging of the quartz tube was identified [25] (Figure S1).

### 2.2. The Characterization and Measurement of Material Properties

**Figure 1:**
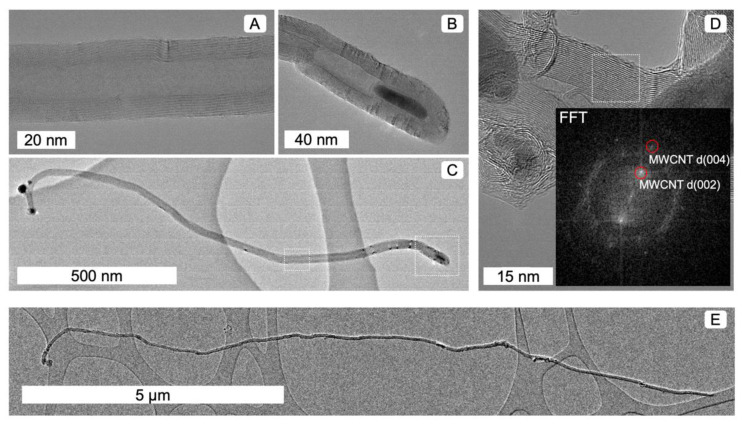
High resolution transmission electron microscope images showing (**A**) multiple walls and (**B**) the presence of internal catalyst particles in (**C**) long MWCNTs. (**D**) FFT analysis confirmed d(002) and d(004) line spacings. MWCNT lengths (**E**) reach over 10 μm in this case.

High resolution transmission electron microscopy (HRTEM) was used to characterise the as-grown samples (Figure 1) using a FEI Talos 200X (FEI, Hillsboro, OR, USA) Transmission Electron Microscope (TEM) in high-resolution TEM mode, operating at 200 kV. The TEM samples were prepared by dipping holey carbon TEM grids into CNT powders. Fast Fourier transforms (FFT) of selected images were obtained to determine the materials’ atomic structures.

**Figure 2:**
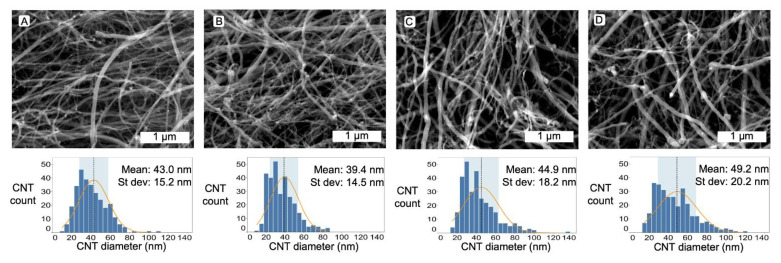
Representative scanning electron microscope images of carbon nanotubes made from (**A**) control with zero PS, (**B**) PS 1 wt%, (**C**) PS 2 wt% and (**D**) PS 4 wt%. Along with histogram data for each sample showing mean and standard deviation for each sample.

Scanning electron microscopy (SEM) using a JEOL 7800F FEG (JEOL, Akishima, Tokyo, Japan) was used to corroborate the presence of MWCNTs (Figure 2). A small fraction of each sample was suspended in 3 mL ethanol, and 100 µL of the suspension was dried on the surface of a clean silicon wafer for imaging. The SEM was used at an operating voltage to 5 kV or below, with a working distance of ca. 10 mm. Diameters were measured using ImageJ [26].

**Figure 3:**
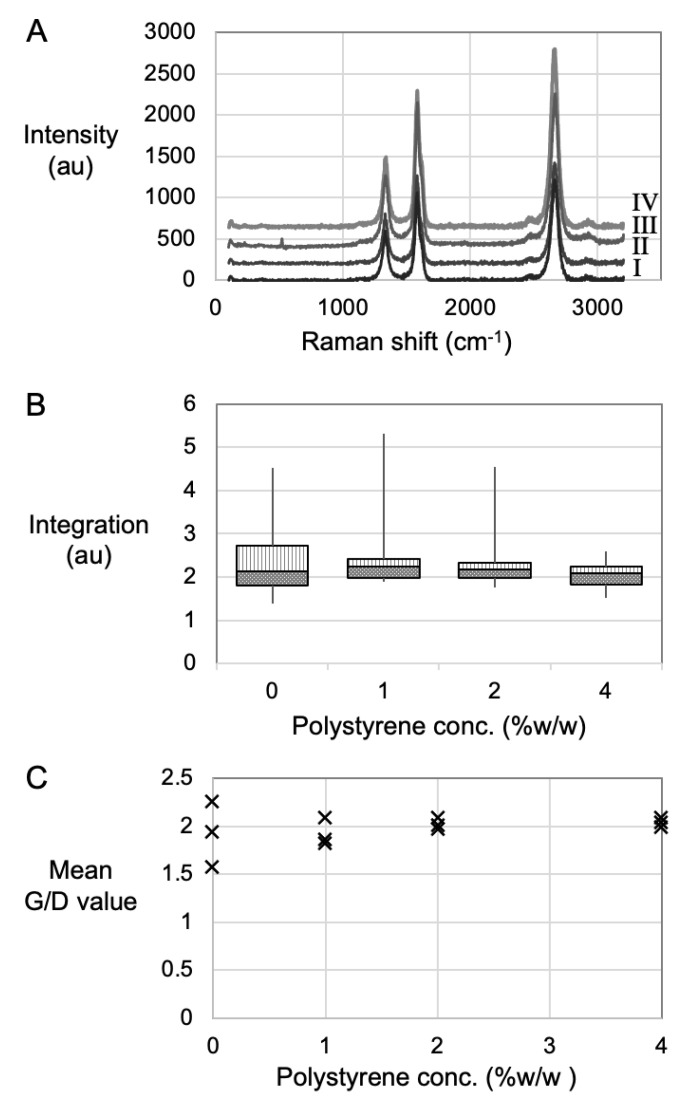
Raman data showing (**A**) typical spectra stacked from (I) Control, (II) PS 1 wt%, (III) PS 2 wt% and (IV) PS 4 wt%. (**B**) Box plot from line integration between 1400–1475 cm^−1^, and (**C**) Mean G/D values. All spectra acquired using 633 nm laser.

A Renishaw inVia Raman microscope (Renishaw plc, Miskin, Pontyclun, UK) using a laser at 633 nm wavelength and 5% beam power was used for data acquisition between 100 cm^−1^ and 3200 cm^−1^ Raman shift. The laser beam was focused by maximizing the G-peak intensity to confirm best z-height alignment of the beam between sample and detector. For each CNT sample, a Raman spectrum was acquired in three separate locations. All G/D values for that series were then averaged and reported along with the maximum G/D for that series and plotted in Figure 3. The Raman spectra also show background intensity in the region from 1400 cm^−1^ to 1475 cm^−1^ that is due to fluorescence in amorphous carbon [27]. Line integration of the background intensity between that range was plotted to compare amorphous carbon content (Figure 3C). For each reaction condition, the individual CNT samples were probed in at least three separate locations in order to both overcome variance within a single sample and to create a significant quantity of data for comparative analyses between series.

Thermogravimetric analysis (TGA/DTA) (TA Instruments, Stamford Avenue, Cheshire, UK) [28] of the CNT samples was used to determine the MWCNT product yield by using ca. 10 mg of sample placed in a platinum pan and heating under active air flow up to 800 °C. The ramp rate was 5 °C/min and hold time was 30 min at 765 °C. The sampling interval was set for 3 s. MWCNT wt% is determined as the complete weight loss after full oxidation at 800 °C in air using TGA [29] (Equation (1)).

### 2.3. Preparation of CNTs for Testing Voltage Drop

Due to presence of residual iron catalyst witnessed in the HRTEM images, an acid wash was used to remove excess iron to establish the CNT voltage drop. The oxidising acid wash, using equimolar HNO_3_ + H_2_SO_4_ reflux (70 °C, 24 h), stripped away the amorphous carbon in addition to helping clear out the graphitic “onion” layers from the residual catalyst materials that would otherwise hinder iron from being digested [30,31]. Note that the acid wash may have inadvertently damaged and etched some of the MWCNTs, thus increasing the measured resistance. However, it was important to prioritise iron removal, given its potential influence in electrical contact measurements.

### 2.4. Device Preparation and Measurement

Thin films were prepared using the “bucky-paper” [32] technique to the measure electrical conductivity of acid washed samples. After the acid wash, the CNTs were suspended in isopropanol and CNT films were made using vacuum filtration [33]. The CNT films were dried at 80 °C for 3 h prior to testing and use.

Electrical resistance values were derived using Ohm’s law based on measured values of voltage drop at constant current in a range of values between 0 to 100 mA. The samples were measured along a 2 cm separation for all samples to ensure consistent path length. 

The carbon nanotube cables were comprised of CNT powders firmly packed into the sheath of heat shrink tubing. Copper wire was inserted into the CNT wire ends and compressed to ensure maximum contact with the Cu lead prior to heat shrinking the outer sheath. The Cu leads were then used to crimp into pins and inserted into retail purchased RJ45 connectors (RS Components, Corby, UK) for testing as ethernet cable. 

Quantification of the CNT ethernet cable was conducted using iPerf3 (iPerf3 is principally developed by ESnet/Lawrence Berkeley National Laboratory. It is released under a three-clause BSD license). The cable was directly connecting two computers, both running the same Windows 10 OS update, going from a Realtek 8125B 2.5 G LAN adapter (Realtek Semiconductor Corp., Hsinchu, Taiwan) capable for two-way traffic (server) to an Intel Killer E3100X LAN adapter (Intel, Santa Clara, CA, USA) as the client; both were capable of transfer speeds up to 2500 Mbps, ensuring that the CNT cable could perform without being bottlenecked. A standard 10 s/10 run test was performed to both authenticate the data transfer and measure the transfer speeds from the server (uplink) to the client (downlink). The test was repeated for statistical accuracy and the results recorded for further discussion.

### 2.5. Life Cycle Assessment(LCA) Methodolgy, Assumptions, and Boundary Conditions

An LCA was conducted using Simapro 9.1 with Ecoinvent 3.6 as database using the method “IPCC 2013 GWP 100a (incl. CO_2_ uptake) V1.00”. In the following LCA, the CO_2_ emissions (Global Warming Potential) are expressed as CO_2_ equivalent (CO_2_ eq.) for the extraction, production, transport, use and end of life phases of CNT and copper wires. They were compared in the use case of electrical wiring in a standard Boeing 747-400 aircraft. This study includes analyses of both the environment impact of the manufacturing of CNT wires compared to Cu wire, and of the effect these wires have over the lifespan of a 747 in terms of CO_2_ generation from fuel consumption.

Assessing the potential environmental impacts of CNT manufacturing based on life cycle assessment required the following assumptions: the mass of 141 miles [34] of copper wire is 1519.6 kg (Supplementary information); copper ore extraction was conducted in Spain; copper wire manufacturing is done in the UK within a 100 km radius of London. The mass of 141 miles of CNT wire was 356.2 kg for the same cross-section as the copper wire [35]. The raw materials for the CNTs were produced locally (within a 300 km radius of Swansea) and the CNTs were manufactured in the UK (using energy from the average UK electricity mix). The CNTs were produced on a laboratory scale as per the methods described herein; this is because no large scale or industrial process uses the LIR model for comparative purposes at this time, and a full study of a scaled reaction process is beyond the scope of this work.

To study the effective change in CO_2_ emissions from an aircraft utilising CNTs wires over Cu wires during its use phase required the following assumptions: an aircraft equipped with CNT wire will be 1163.4 kg lighter. The lifespan of a Boeing 747-400 is approximately 100,000 h of flight time with an average speed of 900 km/h. Only the CO_2_ emissions generated during the use phase of the aircraft were considered in this analysis; accounting for the CO_2_ emissions resulting from the manufacture of the aircraft is beyond the scope of this work.

## 3. Results and Discussion

### 3.1. Material Characteristics Determind Using Microscopy

Using high resolution transmission electron microscopy, we observed the presence of both carbon nanotubes and residual metal catalyst particles (Figure 1). The number of walls ranged between 18 and 52, with CNT diameters ranging from 18 to 45 nm. There was no apparent trend associated with PS loading (within the small number of TEM images counted). Several MWCNTs displayed closed caps, and some had several catalyst particles within a single CNT (Figure 1B). 

The longest single CNT observed was 13.7 µm (Figure 1E), although longer CNTs may exist. The 0.34 nm spot in the FFT is due to the d(002) lattice spacing associated with multiple graphitic walls. This is significant because it further verifies the presence of nanotubes based on their crystallography. Additionally, the 0.17 nm spot is the d(004) spacing from the second order diffraction of the d(002) lattice. However, it is more challenging to differentiate iron oxide compounds such as Fe_2_O_3_, Fe_3_O_4_ and FeOOH from FFTs, as they have very similar spots/d-spacings (Figures S2–S4).

Using scanning electron microscopy (SEM), we easily identified CNTs based on their tangled nature and long morphology (Figure 2). The catalyst content could also be identified due to higher charging density around the Fe and FeC structures. Some of the catalyst could be seen within the CNT structures, further confirming nanotube formation due to the presence of hollow cores. In some cases, the catalyst could be seen as high intensity bright spots, both along the inside walls of the CNTs and at the ends.

Most of the CNTs displayed a relatively tortuous path once deposited on the SEM stubs for imaging. In the control samples, the average diameter was found to be 43.0 nm +/−15.2 nm (Std. Dev). Interestingly, at low PS (1 wt%) concentration, the CNT diameters decreased with a concurrent smaller distribution, resulting in MWCNTs with 39.4 nm +/−14.5 nm average diameter. However, increased PS content led to increased diameters and greater standard deviations. The diameter increase can be accounted for by the increased carbon concentration in the feedstock.

### 3.2. Material Characteristics Determined Using Spectroscopy

Raman spectroscopy is commonly used to measure and characterise the fingerprint peaks associated with MWCNTs (Figure 3A). It can also be used to make comparative analyses of amorphous carbon contents (Figure 3B) between samples. More importantly, resonant Raman spectroscopy is used to quantify the quality of the MWCNTs by direct comparison of G- and D-peak intensities (Figure 3C). 

The representative spectra for all four conditions exhibited G- and D-peaks associated with the presence of MWCNTs (Figure 3A). In the case of MWCNTs, the G-peak (also referred to as G mode, or Graphitic mode) appeared in the 1500–1600 cm^−1^ range due to the tangential displacement of C–C bond stretching, effectively indicating the density of the sp^2^ hybridized carbons atoms that existed in the CNT lattice, and along the circumferential direction of the nanotube. However, the D-peak, or Disorder mode, observed at 1290–1330 cm^−1^, represented the conversion of carbon centres from sp^2^ to sp^3^ hybridisation states, as can occur due to a break in the symmetry of the graphite plane [36]. Therefore, comparing the peak heights of the G- and D-peak effectively quantified the graphitic versus the defective carbon atoms within the CNT lattice. The D peaks intensities must be taken with respect to the G peak intensities. When the D peaks are high, it indicates a large portion of sp^3^ carbon atoms. sp^3^ carbon atoms can arise for several reasons relating to the broken symmetry of the graphitic structure. For this reason, the D-peak is used to identify defects in the CNT structure; it is not that the CNTs are themselves defective, but that they have imperfections within their body. A third fingerprint peak is also evident in Figure 3A; this G′ (G prime) peak corresponds to disorder-induced carbon features arising from finite particle size distribution or lattice distortions in the CNTs. Moreover, the presence of G’ peak further indicated the presence of multiple walls, which was to be expected for samples of multiwalled carbon nanotubes.

The spectral background intensity in the range 1400–1475 cm^−1^ occurred due to the fluorescence of amorphous carbon and line integration in that region was used to compare amorphous carbon concentrations between samples [27,37]. A box plot [38] (Figure 3B) of the line integration values indicates smaller and narrowing range with higher PS loading. As small integration values indicate less amorphous carbon content, we concluded that amorphous carbon decreases as a consequence of increased PS loading.

The G/D value indicated quality in bulk samples, since a large G-peak relative to the D-peak indicates a strong resonance condition of sp^2^, graphitic carbon. [39] Comparisons between reaction conditions could be made in terms of range variance and minimum versus maximum values for each reaction condition. The mean G/D values for the control group suggested that, on average, this process yielded high quality materials, but they ranged from 1.5 to 2.3, suggesting they were more heterogeneous samples compared to the PS 4 wt% condition that had an average range from 1.9 to 2.1. Moreover, the PS samples generally displayed a tightening of data points which suggested greater homogeneity. The G/D values improved with a tightened range too, indicating that higher quality materials had been created by the use of PS, whereby the maximum G/D value was recorded using the PS 4 wt% (Table 1). Moreover, the increase of PS concentration may not have been detrimental, based on the steady measurement of the mean value at 2.0, even after the increase.

### 3.3. Material Properties Determined Using Mass Balance and Thermogravimetric Analysis

**Figure 4:**
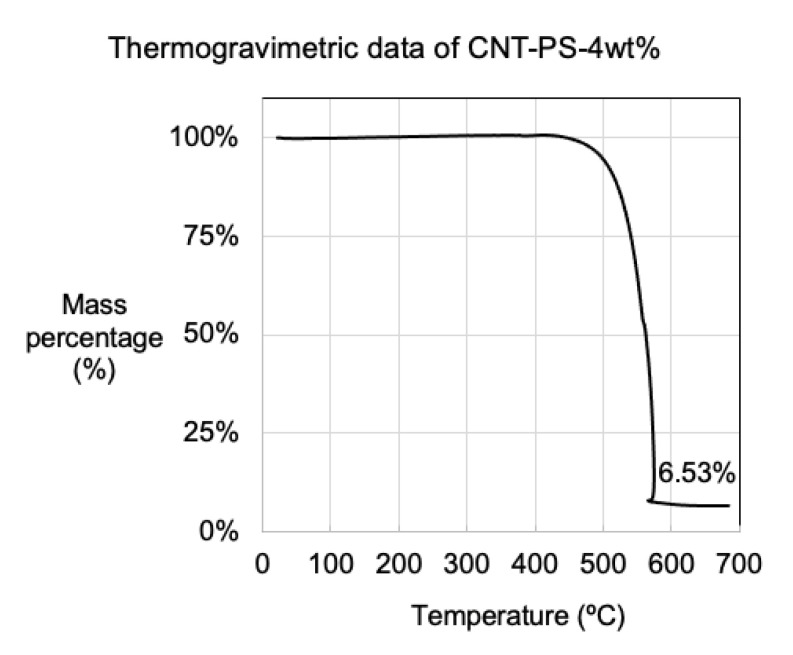
Thermogravimetric data of CNT growth from polystyrene 4 wt% loading, residual mass found to be 6.53%, composed of iron oxide residue from catalyst precursor.

The solid product was weighed using mass balance and used to determine MWCT percentage yield according to Equation (1). TGA data based on the oxidization, and subsequent mass loss at temperatures between 400–600 °C, were characteristic of MWCNTs (Figure 4). The residual mass in the TGA pan was the oxidised catalyst material, typically orange in colour and represented by a residual mass percentage which was used to calculate MWCNT percentage yield (Equation (2)). For example, using 4 wt% of PS, 130 mg was recovered from the reactor. A TGA test showed 6.53% to be residual catalyst, equating to a MWCNT mass of 121.55 mg, which, in turn, corresponded to a CNT percentage yield of 14.2% (Equation (2)). All the values were tabulated and averaged to determine the average CNT product yield (Table 2). Since we were dealing with catalytic processes where some reactions showed outstanding results compared to the norm/average, we have also reported the maximum percentage yields. Though not the norm, they represent the best-case scenario. Based on TEM observations, we assumed that negligible quantities of amorphous carbon were present; this assumption was reinforced by the line integration study of the Raman spectra. 

Based on the aforementioned observations that increased PS loading, and therefore higher carbon density samples, yield MWCNT with wider walls (Figure 2) with lower amorphous carbon content (Figure 3B) and higher quality due to higher maximum G/D values (Figure 3C), it was concluded that the carbon from the PS had become carbon in the MWCNTs. This correlates with the both the Baker model [40] of carbon fibre growth and the Puretzky model [41] of CNT growth, whereby as more carbon enters the reaction, more walls are created.

(1) $$CNT\;percentage\;yield=\frac{\left(Mass_{Product}\right)-\left(Mass_{Fe\;residue}\right)}{Mass_{Theoretical\;yield}}\times 100$$

(2) $$CNT\;percentage\;yield=\frac{130.00\;\left(\mathrm{mg}\right)-8.45\;\left(\mathrm{mg}\right)}{849.23\;\left(\mathrm{mg}\right)}\times 100=14.31\%$$

### 3.4. General Mechanism of Carbon Nanotube Growth

The CNT growth mechanism comprised a multistep process involving the formation of catalysts, the decomposition of carbon sources, and the reconstitution of carbon to a nanotube structure. This process began with the in situ decomposition of ferrocene, i.e., dissociation of the cyclopentadienyl rings sandwiched about the Fe core to render a mixture of Fe_2_O_3_ and Fe_3_O_4_ catalytic nanoparticles. The oxide form was anticipated because the reaction chamber was not first pumped down; therefore, it is likely that some amount of oxygen was resident in the chamber despite the fact the system had been flushed with concentrated argon in advance of each reaction.

The iron agglomerated and formed different sized structures ranging from nanoparticles to microparticles. These iron particles acted as catalytic surfaces known to catalytically cleave the C-H bonds in hydrocarbon reactants such as cyclopentadienyl, polystyrene plastics, and toluene. The lifetime and activity of the catalyst was maintained by using a constant flow of hydrogen in the gas stream that simultaneously reduced the metal, making it active for carbon cracking, and attracted errant carbon moieties that might otherwise have saturated the catalyst, leading to coking and eventual catalyst poisoning. Complete control over CNT products is highly challenging because of catalysts are highly structure-sensitive [42] materials, and even slight variance from the optimum condition can lead to drastic changes in the product, as determined by volcano plots. A key struggle with CNT growth is the inverse relationship between sample yield and quality—it is consistently one or the other.

A mechanistic understanding of carbon nanotube growth suggests that carbon in the form of C2 [43,44,45] enters the catalyst lattice and saturates the molten metal crystal. Following saturation within the catalyst, carbon precipitation occurs at a growth facet, typically a high energy facet such as <111>, exiting the catalyst in graphitic tubular form. The tubular morphology of the carbon is physically bounded by the outer edges and outer diameter of the catalyst particles and the graphitic form is adopted because of the low entropy state of graphite.

### 3.5. Contribution to CNT Growth from TOLUENE

Toluene decomposition compliments CNT growth from plastics in two distinct ways. Firstly, liquid phase dissolutions allows for sonication and, though resulting cavitation, assists the PS decomposition by decreasing the PS molecular weight [46]. Then, in the gas phase (at the conditions used for CNT growth), toluene readily forms C2 units, leading to CNT nucleation. CNT nucleation is notably a function of hydrocarbon thermal decomposition to suitable fragments (C2 units). C2 formation from thermal decomposition of toluene [47,48,49] can occur via several pathways. For example, toluene can decompose either through benzyl radical (Equation (3)) or phenyl radical (Equation (4)) formation; however, the former dominates at the temperatures applied in the present study. The benzyl radical undergoes subsequent decomposition to form C5, C3, and C4 units, that can, in turn, decompose further; see Equations (5) and (6). Toluene decomposition leads to the formation of C2, C3 and C4 units that decompose either in the gas phase due to temperature or via radical attack, or on the catalyst particle surface, to render the C2 fragments associated with nanotube growth. Moreover, the highly active C2 units or radicals mentioned herein can also initiate and advance PS decomposition.

C_6_H_5_CH_3_ → C_6_H_5_CH_2_* + H* (3)

C_6_H_5_CH_3_ → C_6_H_5_* + CH_3_* (4)

C_6_H_5_CH_2_* → C_5_H_5_ + C_2_H_2_ (5)

C_6_H_5_CH_2_* → C_3_H_3_ + C_4_H_4_ (6)

### 3.6. Contribution to CNT Growth from Polystyrene

PS is a conjugated aromatic polymer structure that can be decomposed by scissioning the conjugated chain into the styrene monomer units via H* attack [50]. H* originates from several sources, both in the gas and liquid phase. For example, liquid phase decomposition of toluene (Equation (3)) liberates H*, especially during the sonication used in the mixing of the liquid or at elevated temperatures. The temperature in the CVD furnace vaporizes toluene, resulting in further H* release which compliments H* originating from the thermal splitting of hydrogen in the gas flow. Further H* can be formed at elevated temperatures by catalytic cracking of hydrocarbon over an iron oxide catalyst [51]. Even without a catalyst, thermal cracking of the polymer produces an aromatic product state consisting of high concentrations of styrene (~50–79 wt%), together with the styrene dimer and trimer and other aromatic compounds including toluene, xylene, and alkylated benzenes [52]. There is a complimentary cascade of chemical reactions induced by various pathways to create H* that assist in the decomposition of polystyrene to styrene monomers. Once decomposed to styrene monomer or a similar hydrocarbon, they are further decomposed to C2 units, allowing CNT growth to occur.

### 3.7. Contribution to CNT Growth from Cyclopentadienyl Rings

Carbon from the cyclopentadienyl could also contribute to the carbon of the CNT [53]. Cyclopentadiene moieties, sandwiching iron in the case of ferrocene, tend to exhibit relatively high thermal stability on account of the strong binding affinity between those groups and the internal metal atoms [54]. Therefore, complete decomposition of the ferrocene complex was only expected to occur in zone two, where the temperature for growth was set at 780 °C. First, the cyclopentadienyls disassociated from the iron core and underwent further decomposition in the presence of the newly formed catalysts nanoparticles. Reaction products such as methane and ethane readily formed [55]; these compounds are widely used as carbon sources for CNT growth. Therefore, carbon from the cyclopentadiene could also potentially form part of the CNT product. In all reactions, the ferrocene concentration was fixed at 5 wt% of the mass of solvent and plastic. For example, in the control, in the absence of polystyrene plastic, 41.15 mg of ferrocene was used, thus contributing 26.34 mg of carbon. Note however that the average mass of the control reactions was 89.9 mg (Table 2, Figure S5). The carbon from the cyclopentadiene only consisted of ~30% of the average CNT mass in the control reactions, indicating the carbon from the cyclopentadiene could only form part of the CNT product; the remaining carbon must have originated from the solvent, or, when PS was added, from the PS too. 

### 3.8. Carbon Nanotube Devices Measurement and Application

**Figure 5:**
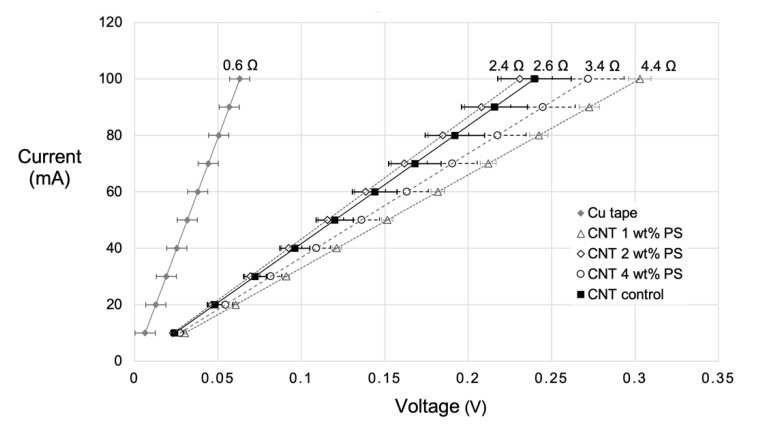
Voltage measurements of acid-washed carbon nanotube samples as compared with commercial copper tape.

In all cases, the voltage increased linearly with current, indicating ohmic resistance of the CNT films. As a reference, the electrical resistance of a retail piece of copper tape was measured by the same procedure. The electrical resistance of the CNT samples was found to be in the range of 2.4 to 4.4 Ω. This is two orders of magnitude higher than that of the copper reference sample. On average, the use of PS increased electrical resistance (Figure 5). However, the best conductivity performance came from sample PS-2 (2 wt% *w*/*w*), i.e., 2.4 Ω compared to R = 0.6 Ω with the control. Although these values are generally higher than that of copper, the mass difference between carbon and copper make these materials an attractive alternative to copper once the I^2^R losses are improved, especially since their lightweight nature is critical. Moreover, it is likely that our attempt to remove contributions from catalyst particles [56,57] (used as catalyst) by virtue of the acid wash may inadvertently have resulted in the poor electrical properties. The oxidizing acid wash may have created additional sidewall defects that ultimately hindered electron transport and this may be why no apparent trend in electrical performance was noted regarding PS loading in the feedstock. 

**Figure 6:**
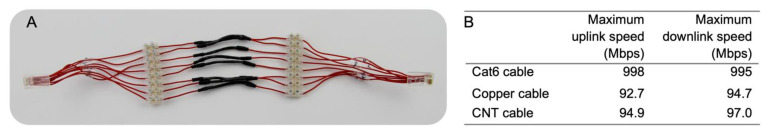
Photograph showing the CNT ethernet cable made using polystyrene-toluene feedstock (**A**), and the ethernet speed results using three devices, one (Cat6) commercial device and two lab made devices using CNTs or CU wire as active transmission component (**B**).

By quantifying the uplink and downlink speed of a CNT ethernet cable (Figure 6A) using iPerf3, we concluded that the MWCNT wires were capable of data transfer rates of at least 99 Mbps. Based on our findings, the Cu wire gauge used, as well as the RJ45 connectors, were holding back the potential of the CNT wires, as determined by the fact Cu wires, once connected to identical RJ45 connectors, also maxed out at 94.7 Mbps (Figure 6B). Industrial grade RJ45 connectors certified for the CAT7 standards coupled with higher quality, thicker gauge copper wiring as well as better assembly and crimping would likely help negate the bottleneck. As it stands, the CNT ethernet cables can meet to the rated uplink and downlink speeds of the CAT5 standard, with results saturating at 100 Mbps (Video S1), as has been seen previously [58]. These speeds are adequate for UK adoption and Broadband classification, as determined by the UK Government regulator [59].

### 3.9. The Life Cycle Assessment of MWCNT Growth and Wire Production Versus Cu Wire Formation

**Figure 7:**
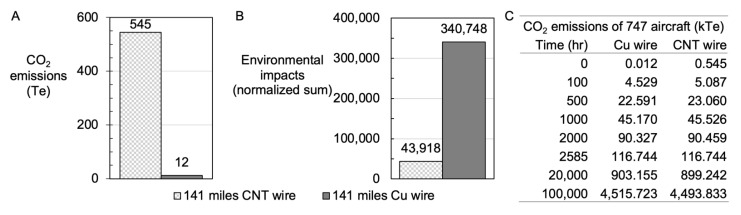
Life cycle analysis comparing (**A**) CO_2_ emissions and (**B**) normalized sum of all environmental impacts between the production of 141 miles of CNTs wires versus Cu wire. Comparative table (**C**) showing the CO_2_ emission (kTe) per copper versus CNT wires.

What is the impact of making CNT wires (lab scale) compared to making an equivalent length of Cu wire from an industrial process? Moreover, what carbon saving could be achieved on account of a lighter aircraft over its typical lifespan, and where is the breakeven point for using CNT wires instead of Cu wire? For every 1 kg of CNT powder, 1.58 kg of CO_2_ is created (Supplementary information), largely due to the electricity requirement for heating [60,61]. The electricity sources available in the UK did not make a significant impact on decreasing the CO_2_ generation (Supplementary information). Using the CNT powder to make 141 miles of cabling, 356.2 kg of CNTs resulted in CO_2_ emissions of 545 metric Tonnes (Te), compared to making 1519.6 kg of copper wire, which generated 12 Te of CO_2_; this is nearly 45 times more CO_2_ just from the manufacturing of CNT cables compared to Cu wires (Figure 7A). However, once economies of scale such as Wright’s Law [62,63] have been considered, we anticipate CNT cable manufacturing on a larger scale to decrease CO_2_ emissions [64]. 

Other environmental factors aside from CO_2_ emissions exist, such as (but not limited to) freshwater eutrophication, freshwater ecotoxicity, marine ecotoxicity and stratospheric ozone depletion. When the normalised sum [65] of these impacts is compared between CNT (at lab scale) and Cu wires, one can note an approximate eight-fold increase in the negative impact from Cu wire manufacture (Figure 7B). 

### 3.10. The Life Cycle Assessment of Use Case of MWCNTs in a Boeing 747-400 Aircraft

Using CNT wires would make a Boeing 747-400 series 6% lighter; this saving would improve fuel efficiency, such that after just 2585 h of flight-time, overall CO_2_ emissions would decrease comparatively (Figure 7C). This savings could be improved once the wire manufacturing process were scaled by virtue of scaling laws in manufacturing proficiencies. Note that almost 99% of the CO_2_ eq. emissions are CO_2_ emissions (of the 4520 kTe of CO_2_ eq. emitted by the plane, 4450 kTe are CO_2_ emissions). Due to the lightweight nature of CNT wires compared to heavier Cu wires, over the life span of the aircraft (100,000 h), the use of CNT wires decreases the carbon footprint by 21 kTe per plane. Moreover, given the fact that 694 craft were delivered between 1989 and 2009 [66] a total of 14,574 kTe CO_2_ reduction could be projected for the 747-400 fleet. This projection demonstrates how the use of lightweight carbon wire technologies could create positive impact towards solving global CO_2_ grand challenges.

## 4. Conclusions

Multiwalled carbon nanotubes (MWCNTs) were grown using liquid injection chemical vapour deposition (CVD) at 780 °C using ferrocene catalyst particles to obtain carbon from ferrocene, toluene and polystyrene (PS) at various PS concentrations, i.e., from 1 to 4 wt% (*w*/*w*). Samples were characterized using scanning electron microscopy (SEM) and transmission electron microscopy (TEM). MWCNT diameters were found to increase with greater PS concentrations due to carbon from PS incorporating into the MWCNTs. Quality was measured using Raman spectroscopy. The maximum Raman G/D values both increased and the range of mean values narrowed due to the presence of higher quality products, with greater homogeneity at greater PS concentration. This synthesis method improved MWCNT quantity without incurring a measurable loss in quality. Due to the electrical nature of MWCNTs, a MWCNT ethernet cable was produced, which found to have ~99.5 Mbps uplink and downlink speeds, i.e., comparable to those of Cu wires of similar diameter. A life cycle assessment (LCA) of the MWCNT wires made using PS suggested that the electricity powering the CVD furnace represented the largest impact. Moreover, the LCA determined that over the lifespan of a single Boeing 747-400, the use of MWCNTs wires would decrease CO_2_ production by 21 kTe due to the lightweight nature of MWCNTs which is projected to reduce 14,574 kTe CO_2_ footprint across the entire fleet of 747-400 aircraft. This projection demonstrates how a plastic circular economy making lightweight energy transmission carbon cables can impact global grand challenges.

## 5. Patents

Two patents have been filed from this work. A.H. and A.O.W. filed: PROCESS FOR REUSE OF PLASTIC THROUGH THE CONVERSION TO CARBON NANOMATERIALS United States Patent Application 20190375639; T.Y. and A.O.W. filed: CABLES AND METHODS THEREOF United States Patent Application 20210158995.

## Figures and Tables

**Table 1:** Mean and maximum G/D values obtained for various carbon nanotube samples grown with incrementally higher concentrations of polystyrene feedstock.

	PS Concentration (wt%)			
	0	1	2	4
Mean G/D value	1.9	2.0	2.0	2.0
Maximum G/D value	2.3	2.3	2.3	2.5

**Table 2:** Mass data and percentage yield results for CNTs grown using polystyrene.

Sample	Mass of Carbon Reactant (mg)	Average CNT Product (mg)	Maximum CNT Product (mg)	Maximum CNT Yield (%)
Control	815.87	89.80	92.80	11.37%
1 wt% PS	824.20	80.47	89.36	10.84%
2 wt% PS	832.57	100.36	107.83	12.95%
4 wt% PS	849.23	106.58	121.55	14.31%

## Data Availability

The data presented in this study are available on request from the corresponding author.

## Supplementary Materials

The following are available online at https://www.mdpi.com/article/10.3390/nano12010009/s1, Figure S1: MWCNT yield versus order of the reaction, Figure S2: FFT of HRTEM images (MWCNT)., Figure S3: FFT of HRTEM images (local area), Figure S4: FFT of whole HRTEM image, Figure S5: Screenshot of mass balance calculations for reactants showing relevant carbon content, Video S1: Video showing the iPerf3 testing of ethernet speeds of CNT ethernet cable.
