# Supplementary material for: On the Use of Carbon Cables from Plastic Solvent Combinations of Polystyrene and Toluene in Carbon Nanotube Synthesis
Source: Nanomaterials (Basel). 2021 Dec 21;12(1):9. doi: 10.3390/nano12010009 (PMC8746690; doi:10.3390/nano12010009)
Supplement: Supplementary file 1 [file nanomaterials-12-00009-s001.zip › nanomaterials-1465201-supplementary.pdf]

nanomaterials

Supplementary Information

On the Use of Carbon Cables from Plastic Solvent Combinations of Polystyrene and Toluene in Carbon Nanotube Synthesis

Alvin Orbaek White [1,2,*], Ali Hedayati [1,3], Tim Yick [1], Varun Shenoy Gangoli [1], Yubiao Niu [4], Sean Lethbridge [4], Ioannis Tsampanakis [1], Gemma Swan [1], Léo Pointeaux [1,5], Abigail Crane [1], Rhys Charles [5], Jainaba Sallah-Conteh [1], Andrew O. Anderson [1], Matthew Lloyd Davies [5,6], Stuart. J. Corr [7,8,9,10] and Richard E. Palmer [4]

[1] Energy Safety Research Institute, Swansea University, Bay Campus, Swansea SA1 8EN, UK; alihe@chalmers.se (A.H.) 748963@Swansea.ac.uk (T.Y.); V.S.Gangoli@Swansea.ac.uk (V.S.G.); 958003@Swansea.ac.uk (I.T.); 9901886@Swansea.ac.uk (G.S.); 2124215@Swansea.ac.uk (L.P.); 910979@Swansea.ac.uk (A.C.); 825585@Swansea.ac.uk (J.S.-C.); 934622@Swansea.ac.uk (A.A.)
[2] Chemical Engineering, Faculty of Science and Engineering, Swansea University, Bay Campus, Swansea SA1 8EN, UK
[3] TECNALIA, Basque Research and Technology Alliance (BRTA), Alava Science and Technology Park, Leonardo da Vinci 11, 01510 Vitoria-Gasteiz, Spain
[4] Nanomaterials Lab, Mechanical Engineering, Faculty of Science and Engineering, Swansea University, Bay Campus, Swansea SA1 8EN, UK; Yubiao.Niu@Swansea.ac.uk (Y.N.); 906934@Swansea.ac.uk (S.L.); R.E.Palmer@Swansea.ac.uk (R.E.P.)
[5] SPECIFIC, Materials Science and Engineering, Faculty of Science and Engineering, Swansea University, Bay Campus, Swansea SA1 8EN, UK; R.Charles@Swansea.ac.uk (R.C.); M.L.Davies@Swansea.ac.uk (M.L.D.)
[6] School of Chemistry and Physics, University of KwaZulu-Natal, Durban 4041, South Africa, RSA
[7] Department of Cardiovascular Surgery, Houston Methodist Hospital, Houston, TX 77030, USA; sjcorr@houstonmethodist.org
[8] Department of Bioengineering, Rice University, Houston, TX 77005, USA
[9] Department of Biomedical Engineering, University of Houston, TX 77204, USA
[10] Swansea University Medical School, Institute of Life Science 2, Swansea University, Singleton Park, Swansea SA2 8PP, UK
* Correspondence: Alvin.OrbaekWhite@Swansea.ac.uk

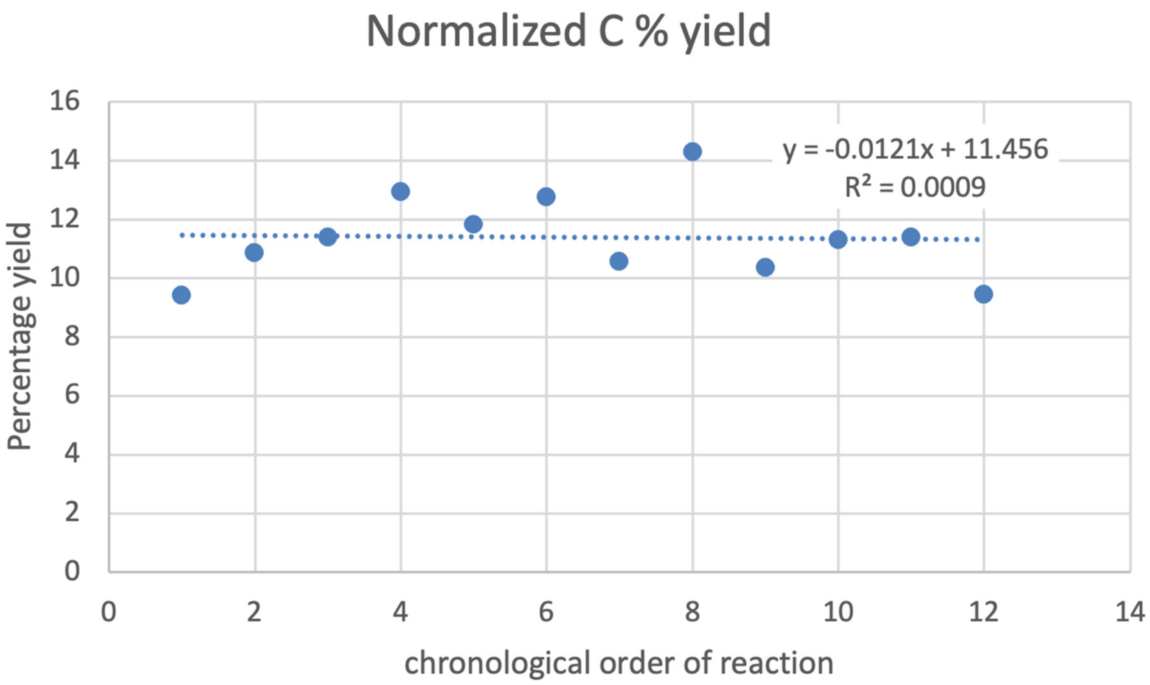

Figure S1. MWCNT yield versus order of the reaction.

Comment: Seasoned veterans growing carbon nanotubes are aware that ageing of the quartz tube, wherein the quartz tube becomes black or the quartz tube develops a carbon layer through subsequent growth, can improve Carbon Nanotube growth through the process of carbothermic reduction. We note that in between each reaction, no cleaning or no chemical cleaning of the quartz tube was carried out. So, all the reactions were carried out on an aged quartz tube, and because of this, we carried out the reactions in a randomised order to mitigate cumulative positive results that compound as the ageing continues, as opposed to being due to the added PS content. Given the fact the trendline is effectively flat we suggest the positive results growth in % yield can be attributed to the actual concentration of polystyrene content and not from the cumulative use, or aging of the furnace. Therefore, aging of the tube is not responsible for the increased percentage yields recorded in this work.

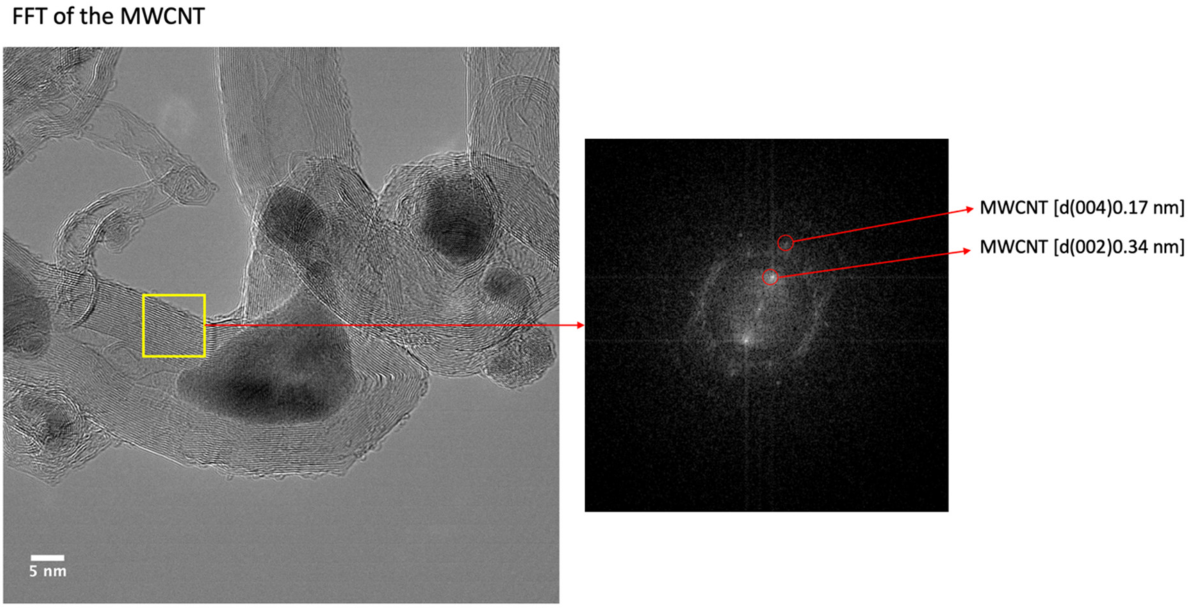

Figure S2. FFT of HRTEM images (MWCNT).

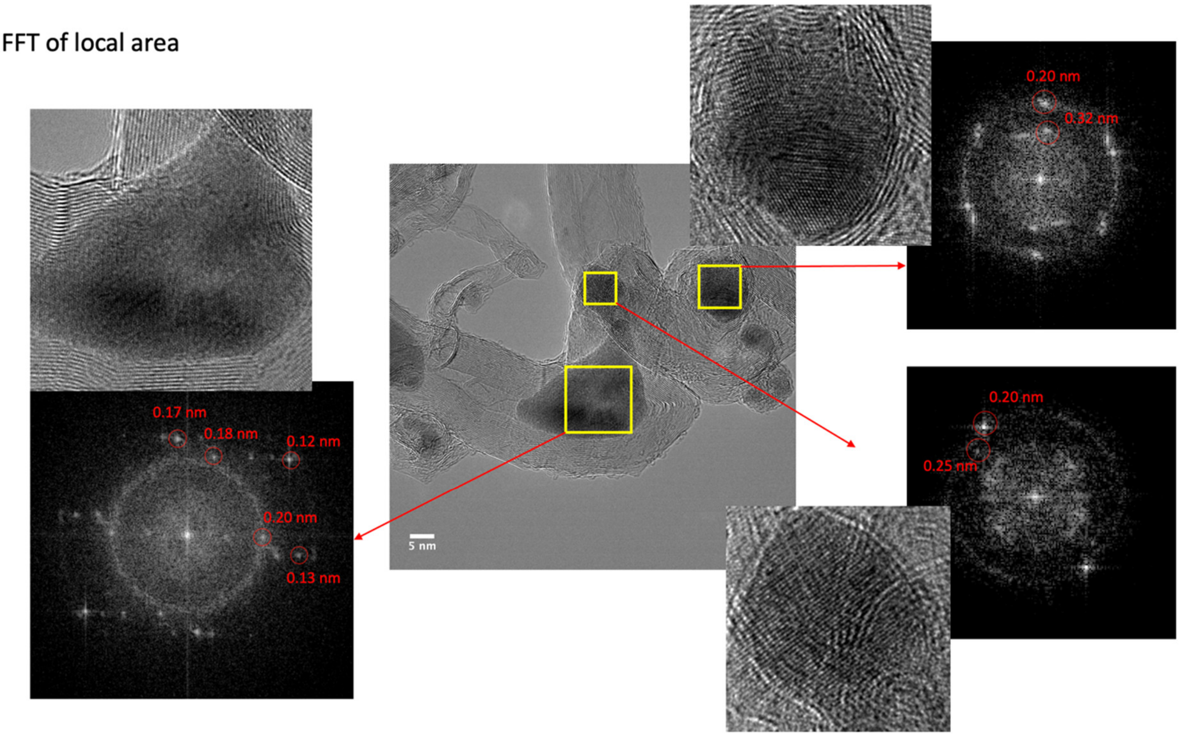

Figure S3. FFT of HRTEM images (local area).

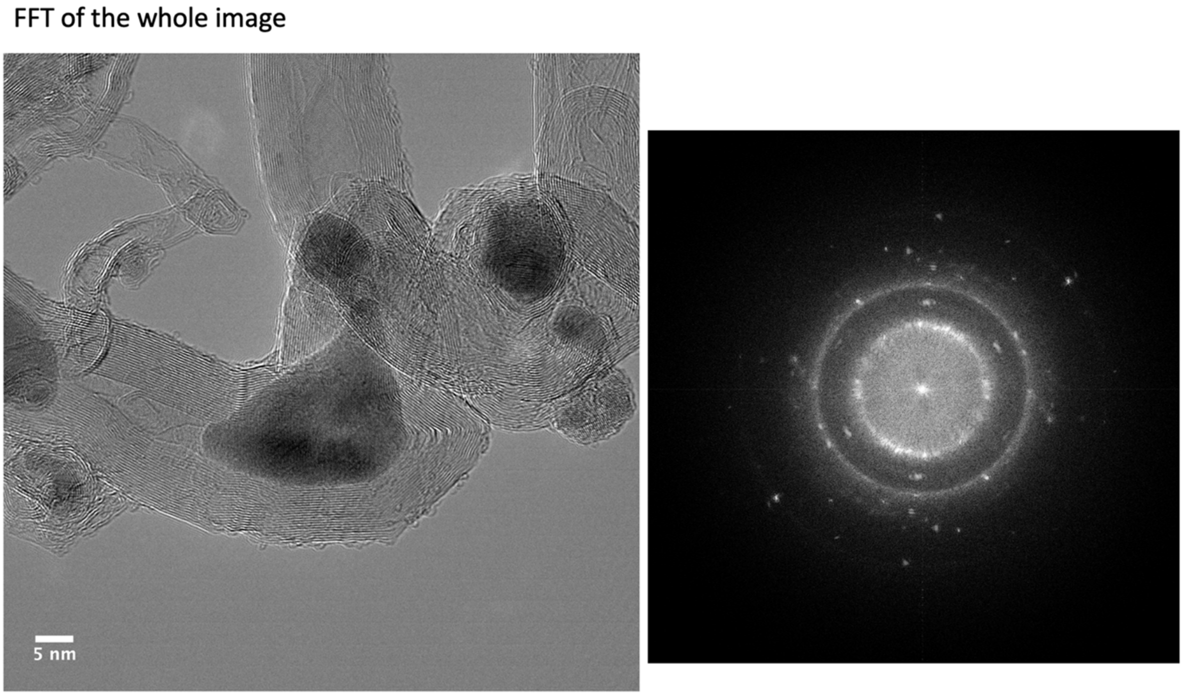

Figure S4. FFT of whole HRTEM image.

**Ferrocene**		AMU		%
Fe	1	55.845	55.845	30.02%
C	10	12.0107	120.107	64.56%
H	10	1.00784	10.0784	5.42%
			**186.0304**	
Fisher			186.04 g/mol	

**Toluene**		AMU		%
C	7	12.0107	84.0749	91.25%
H	8	1.00784	8.06272	8.75%
			**92.13762**	
Fisher			92.14 g/mol	

**PS. MW 6400**		AMU		%
C	8	12.0107	96.0856	92.26%
H	8	1.00784	8.06272	7.74%
			**104.14832**	
Sample #: P2444-S			6400 MW	

Experiment	Control	1 wtPS	2 wtPS	4 wtPS
Ferrocene (mg)	41.15	41.55	42.00	42.80
Toluene (mg)	865.00	865.00	865.00	865.00
PS (mg)	0.00	8.75	17.50	35.00
C' from Ferrocene (mg)	26.57	26.83	27.12	27.63
C' Toluene (mg)	789.31	789.31	789.31	789.31
C' PS (mg)	0.00	8.07	16.15	32.29
total carbon mass (mg)	815.87	824.20	832.57	849.23
total carbon mass (g)	0.816	0.824	0.833	0.849

Figure S5. Screenshot of mass balance calculations for reactants showing relevant carbon content.

Discussion on potential environmental impacts of CNT manufacturing

✈ ✈ *This is what it is written in the Simapro Database Manual about normalization.*

"Many methods allow the impact category indicator results to be compared by a reference (or normal) value. This mean that the impact category is divided by the reference. A commonly used reference is the average yearly environmental load in a country or continent, divided by the number of inhabitants. However, the reference may be chosen freely. You could also choose the environmental load of lighting a 60 W bulb for one hour, 100 km of transport by car or 1 liter of milk. This can be useful to communicate the results to non LCA experts, as you benchmark your own LCA against something everybody can imagine. In SimaPro, there are often alternative normalization sets available. After normalization the impact category indicators all have the same unit, which makes it easier to compare them. Normalization can be applied on both characterization and damage assessment results. PLEASE NOTE: SimaPro does not divide by the reference value (N), but multiplies by the inverse. If you edit or add a normalization value in a method, you must therefore enter the inverted value (1/N)."

Link to the Database Manual: DatabaseManualMethods.pdf available on simapro.com

⚜ Discussion on Fossil CO_2 eq and CO_2 uptake

The only information shared in the Simapro Database Manual about the method used for the CO_2 emissions calculations are the following ones (Method: IPCC 2013 GWP 100a).

"IPCC 2013 is an update of the method IPCC 2007 developed by the International Panel on Climate Change. This method lists the climate change factors of IPCC with a timeframe of 20 and 100 years. 6.4.1 Characterization IPCC characterization factors for the direct (except CH4) global warming potential of air emissions. They are:

• not including indirect formation of dinitrogen monoxide from nitrogen emissions.

• not accounting for radiative forcing due to emissions of NOx, water, sulphate, etc. in the lower stratosphere + upper troposphere.

• not considering the range of indirect effects given by IPCC.

• not including CO_2 formation from CO emissions"

Moreover, they say that "Fossil based carbon" correspond to carbon originating from fossil fuels and "Carbon uptake" correspond to the CO_2 that is stored in plants and trees as they grow.

In our case, most of the CO_2 absorption comes from energy consumption. As the Simapro database does not share much information in this area, it can only be assumed that this uptake is due to the fact that the UK uses incinerators as an energy resource in part. Thus, the reuse of household waste is taken into account in the LCA and appears as CO_2 uptake.

⚜ Discussion on CO_2 eq emissions of an airplane calculations

Here is the list of all the assumptions that I made to calculate the CO_2 emissions of an airplane during its use.

• The weight of copper wire in a plane is equal to 1 519.6 kg. This weight has been determined using the document "Aircraft Electrical Wire" [1] from the company Tyco Electronics. The paper state that the cables, model 55PC, used in Boeing 747 aircraft since 1992, weight 4.5 lbs per 1,000 ft length.

So $141\ miles\ =\ 744{,}480\ ft$

-> $4.5 \times \frac{744{,}480}{1000} = \ 3{,}350.16\ lbs \rightarrow \sim 1519.6\ kg$, this results in a weight saving of 1163.44 kg.

• The weight of CNTs has been determined by dividing the weight of copper wire by the copper density (8.96 g/cm^3) and then multiplicated by the CNTs density (2.1 g/cm^3) [Lu et al. *"Determination of carbon nanotube density by gradient sedimentation"*] [2].

So, $1519.6 \times \frac{2.1}{8.96} = \ 356.16\ kg$

• A Boeing 747-400 fly during its lifespan for approximately 100 000 hours (Here are some websites with information on the life span of the Boeing 747-400: Average Life Expectancy Of A Boeing 747 - Airliners.net, A 747 Aircraft Owned By Idiot Investors Recently Arrived In St Vincent. - Asberth News Network (annsvg.com), The Commercial Aircraft With The Highest Hours And Flight Cycles - Simple Flying) at an average speed of approximately 900 km/h. The value of the average speed of the Boeing has been determined from various information found online. On specialized aircraft websites, it is stated that a Boeing 747-400 flies at a maximum speed of about 930 to 940 km/h and a long range cruise speed of 907km/h (here are some examples of aircraft websites: 1989-2010 Boeing 747-400 | Top Speed, Boeing 747-400 - Price, Specs, Photo Gallery, History - Aero Corner, Airliners.net). Taking into account the take-off and landing phases, I have made the assumption that the aircraft should fly at an average speed of 900 km/h.

• A Boeing 747-400 have an average of 20 000 compression and decompression cycles (A 747 Aircraft Owned By Idiot Investors Recently Arrived In St Vincent. - Asberth News Network (annsvg.com)).

• Using the latest data, I was able to determine that the average length of a Boeing 747-400 flights is 4 500 km. Multiplying the lifetime by the average speed of the aircraft and dividing by the average number of compression and decompression cycles gives the average flight length of a Boeing 747-400.

So, $\frac{100\ 000 \times 900}{20\ 000} = \ 4\ 500\ km$

• An empty Boeing 747-400 has a mass of 183.5 tons, a tank capacity of 217 000 litres and a travel range of 13 500 km (Boeing 747-400 - Price, Specs, Photo Gallery, History - Aero Corner, Boeing 747 Specs: Weight, Length, Range, Wingspan & More (knaviation.net)). With all this information I was able to determine the weight of the plane before it took off for a 4500km flight. By dividing the maximum tank capacity by the maximum travel range and then multiplying by the average length of a flight I get the volume of kerosene needed to complete a flight.

So, $\frac{217\ 000 \times 4\ 500}{13\ 500} \approx \ 72\ 300\ L$, knowing that the density of kerosene is 0.8 g/cm^3,

-> $72\ 300\ \times 0.8\ = 57\ 840\ kg$, adding this mass to the empty mass of the aircraft gives,

-> $183\ 500 + 57\ 840 = 241\ 340$ kg which represents the average weight of a Boeing 747-400 before take-off for a 4500 km flight.

• Knowing now the average mass of the aircraft with copper wires, we can determine its mass with CNT wires.

So, 241 340 - 1163.44 $\approx$ 240 180. kg

• Knowing now the average weight of the aircraft and the distance travelled during its lifetime, it is possible to calculate the tonne-kilometre (tkm) that needs to be fed into the Simapro software to calculate the CO_2 emissions.

So, $241.34 \times 100\,000 \times 900 = 21\,720\,600\,000\ tkm$ for a plane with copper

And $240.18 \times 100\,000 \times 900 = 21\,616\,200\,000\ tkm$ for a plane with CNTs.

• In order to verify the results obtained with Simapro, I tried to find out how much CO_2 was emitted by burning 1 kg of kerosene. According to the website Combustion of Fuels - Carbon Dioxide Emission (engineeringtoolbox.com), 1 kg of kerosene emits 3 kg of CO_2 into the air. Knowing that to travel 13,500 km a Boeing 747-400 needs about 217 000 L of kerosene we can deduce 217 000/13 500 = 16.07 kg of kerosene is required to cover 1 km (I found in the literature that 1 km consumes on average 12.63 kg of kerosene). As mentioned earlier, a Boeing 747-400 will travel approximately 90 000 000 km during its lifetime.

So this gives us,

$$90\,000\,000 \times 16.07 \times 3 \approx 4\,338\,900\,000\ kg\ of\ CO2\ or\ 4\,338.9\ kton\ of\ CO2$$

Or by using the data found online: $90\,000\,000 \times 12.63 \times 3 \approx 3\,410.1\ kton\ of\ CO2$

Knowing that the CO_2 emissions found in the simulation were in the orders of magnitude of 4 500 kton of CO_2 eq (to which just CO_2 contributed 4 450 kton).

References

1. Cinibulk, W. Aircraft Electrical Wire, Wire Manufacturers Perspective, Tyco Electronics (Raychem), **2001**, visited on: aircraft_electrical_wire.pdf (mitrecaasd.org)
2. Lu, Q.; Keskar, G.; Ciocan, R.; Rao, R.; Mathur, R.B.; Rao, A.M.; Larcom, L.L. Determination of carbon nanotube density by gradient sedimentation. *J Phys Chem B* **2006**, *110*, 24371-24376.
