# Supplementary material for: On the Use of Carbon Cables from Plastic Solvent Combinations of Polystyrene and Toluene in Carbon Nanotube Synthesis
Source: Nanomaterials (Basel). 2021 Dec 21;12(1):9. doi: 10.3390/nano12010009 (PMC8746690; doi:10.3390/nano12010009)
Supplement: Supplementary file 1 [file nanomaterials-12-00009-s001.zip › Video S1- Video showing the iPerf testing of Ethernet speeds of CNT Ethernet cable.mp4.html]

 Video S1- Video showing the iPerf testing of Ethernet speeds of CNT Ethernet cable.mp4       

Download

Sign inSign up

[![](https://uc338dfcdf36b72066f27159f2d0.previews.dropboxusercontent.com/p/thumb/ABadaYf49MGUBEUSBQ_Ep7tv0KTtcBOkDNX8Qk6EkDB2K_eSqRhbEKT484oPwgjCBWz-vZrFFdSlHJXElBu1B_5KJVu-vvG4qdbfPdyDhFRCTqmEs0NikV1gh3XXE3iRa8o3QAY6tdTaUBj22EDp-Oej3ISYNMMdNWwEFDo8SzSOgdXsuzDgERzuNjruCU7Hw0UPMLscqKyQ9ezrGg74xJma_XKIbcXs0URTWwe0H1Gal3bwuz3_n1z7_J1TsU10C7F9a6oSOqD3KChZfonl3aJKoNib_VS_6y7WJHszjflRUcN3g_9tfSo-RiDzSwGNoi8/p.jpeg?psid=f3da495d-eb4b-46b6-89cf-4e01353092c1&size=1024x768&size_mode=2)](blob:https://www.dropbox.com/ad0adb5d-5149-474a-b9c7-8b46e4b797b2)

## Get your point across.

Try Capture for freeWatch again

Video Player is loading.

Play Video

This is a modal window.

Beginning of dialog window. Escape will cancel and close the window.

TextColorWhiteBlackRedGreenBlueYellowMagentaCyanTransparencyOpaqueSemi-TransparentBackgroundColorBlackWhiteRedGreenBlueYellowMagentaCyanTransparencyOpaqueSemi-TransparentTransparentWindowColorBlackWhiteRedGreenBlueYellowMagentaCyanTransparencyTransparentSemi-TransparentOpaque

Font Size50%75%100%125%150%175%200%300%400%Text Edge StyleNoneRaisedDepressedUniformDropshadowFont FamilyProportional Sans-SerifMonospace Sans-SerifProportional SerifMonospace SerifCasualScriptSmall Caps

Reset restore all settings to the default valuesDone

Close Modal Dialog

End of dialog window.

You’re watching a preview. To watch the full video, download or add it to your Dropbox.

Video S1- Video showing the iPerf testin…thernet speeds of CNT Ethernet cable.mp4

Loaded: 3.99%

0:00

0:00 / 0:50

   
