# Supplementary material for: On the Use of Carbon Cables from Plastic Solvent Combinations of Polystyrene and Toluene in Carbon Nanotube Synthesis
Source: Nanomaterials (Basel). 2021 Dec 21;12(1):9. doi: 10.3390/nano12010009 (PMC8746690; doi:10.3390/nano12010009)
Supplement: Supplementary file 1 [file nanomaterials-12-00009-s001.zip › Video S1- Video showing the iPerf testing of Ethernet speeds of CNT Ethernet cable.mp4_files/saved_resource.html]

Dropbox Consent


We use cookies to provide, improve, protect, and promote our services. Visit our Privacy Policy and Privacy Policy FAQ to learn more. You can manage your personal preferences in our Cookie Consent Tool.

DeclineAccept All
